# Altered Risk-Based Decision Making following Adolescent Alcohol Use Results from an Imbalance in Reinforcement Learning in Rats

**DOI:** 10.1371/journal.pone.0037357

**Published:** 2012-05-16

**Authors:** Jeremy J. Clark, Nicholas A. Nasrallah, Andrew S. Hart, Anne L. Collins, Ilene L. Bernstein, Paul E. M. Phillips

**Affiliations:** 1 Department of Psychiatry and Behavioral Sciences, University of Washington, Seattle, Washington, United States of America; 2 Department of Psychology, University of Washington, Seattle, Washington, United States of America; 3 Program in Neurobiology and Behavior, University of Washington, Seattle, Washington, United States of America; 4 Department of Pharmacology, University of Washington, Seattle, Washington, United States of America; University of Manchester, United Kingdom

## Abstract

Alcohol use during adolescence has profound and enduring consequences on decision-making under risk. However, the fundamental psychological processes underlying these changes are unknown. Here, we show that alcohol use produces over-fast learning for better-than-expected, but not worse-than-expected, outcomes without altering subjective reward valuation. We constructed a simple reinforcement learning model to simulate altered decision making using behavioral parameters extracted from rats with a history of adolescent alcohol use. Remarkably, the learning imbalance alone was sufficient to simulate the divergence in choice behavior observed between these groups of animals. These findings identify a selective alteration in reinforcement learning following adolescent alcohol use that can account for a robust change in risk-based decision making persisting into later life.

## Introduction

Chronic drug use has been associated with a myriad of persistent consequences for learning and decision making, yet a direct link between these effects has remained largely theoretical. Alcohol is the most commonly abused substance among adolescents [1] and ranks as one of the most harmful [2]. Indeed, individuals who engage in binge drinking at an early age show later increased likelihood of developing alcohol abuse problems [3], [4] and deficits in decision making under risk [5], [6]. We have demonstrated that rats with a history of voluntary alcohol intake during adolescence also make riskier choices than non-exposed animals when they are adults, demonstrating the causal, rather than coincident, nature of this relationship [7]. However, the psychological processes underlying this disrupted decision making remain unknown. In neoclassic economic theory, risk attitude is attributed to the shape of an individual's utility function: the relationship between the objective value of a reward and its desirability (subjective value) [8]. If the growth of subjective value decelerates as objective value increases (i.e., concave utility function), the benefit of two units of a reward is less than twice that of one unit. Therefore, an individual exhibiting this type of utility function would choose a “safe” option of one unit of reward all of the time over a “risky” option of two units half of the time, even though the net *objective* value is the same. As such, the individual is considered risk averse. Thus, increasing the degree to which an individual discounts the subjective value of incremental rewards (more concave utility function) renders them more risk averse. In addition to subjective valuation, economic decision making under uncertainty can also be shaped by reinforcement learning [9]–[12]. This influence may be especially important for decision making where outcomes are variable and therefore deviate from the average expectation (i.e., under risk), promoting ongoing learning [13]. Moreover, it has been proposed that drugs of abuse may exert their behavioral effects through altered reinforcement learning [14]. Retarded learning may limit the use of associative information or lead to perseverative behavior in the face of changing environments, while over-fast learning can be sub-optimal in that it may result in spurious associations and impulsive behavior [15]. In the current work, we sought to determine the psychological constructs, altered by adolescent alcohol use, that underlie this altered risk-based decision making by studying subjective valuation and reinforcement learning processes.

## Results

### Subjective Reward Valuation

**Figure 1:**
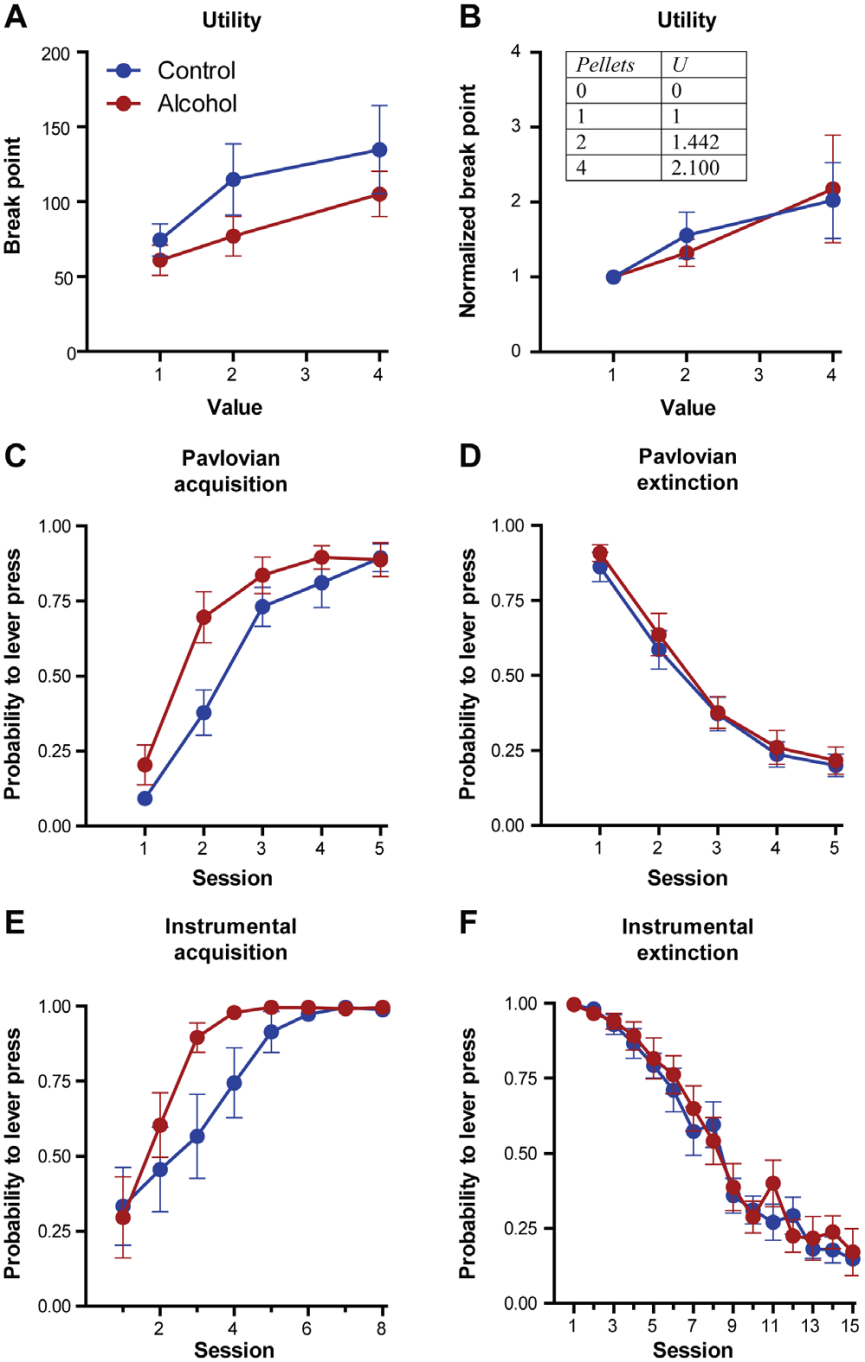
Behavioral data from the progressive ratio task, Pavlovian conditioning, and instrumental conditioning in alcohol-exposed and control animals. (**A**) Mean number of lever presses (break point) on the last completed trial during the progressive ratio task where alcohol-treated (red) and control (blue) animals worked to obtain 1, 2, or 4 sucrose pellets during separate daily sessions. (**B**) Break point data normalized to the 1 pellet session and used for utility estimates (inset). (**C, D**) Acquisition and extinction of Pavlovian conditioning in alcohol-treated (red) and control (blue) groups across all sessions. (**E, F**) Acquisition and extinction of instrumental conditioning in alcohol-treated (red) and control (blue) groups across all sessions. All data are presented as mean ± SEM.

We first evaluated the influence of adolescent alcohol use on subjective reward valuation. Adolescent rats (PND 30–49) were provided with continuous access to a 10%-ethanol or control gelatin prepared with 10% glucose polymers (Polycose) for 20 days. Two months following complete cessation of alcohol access, the amount of work rats (*n* = 12) were willing to perform for a range of reward values was assessed using a progressive ratio task (Figure 1A and 1B). Rats were trained to lever press for sucrose reward and then tested with 1, 2, and 4 sucrose pellets on a work schedule that progressively increased throughout a session. Effort under progressive ratio schedules of reinforcement has been previously established to scale with reward value and to remain stable across multiple sessions [16]–[19]. The maximum work requirement an animal was willing to perform to obtain reward (“break point”) was affected by reward value (main effect of value: *F* [2,20] = 4.16, *P*<0.05) but did not differ between alcohol-exposed animals and controls (main effect of treatment group: *F* [1, 20] = 2.99, *P*>0.05; group×value interaction: *F* [2, 20] = 0.23, *P*>0.05). Animals assigned higher subjective value to larger rewards but the failure to find an interaction effect indicates that the shape of the utility function of the alcohol-exposed group did not significantly differ from controls. Further, a comparison of reaction time data on the progressive ratio task did not reveal a significant main effect of treatment group (*F* [1,20] = 0.01101, *P*>0.05) nor an interaction between value level and treatment group (*F* [2,20] = 0.5965, *P*>0.05). Thus, consistent with our previous report [20] we found no evidence for a general alteration in reward valuation that could account for the altered choice behavior in alcohol-exposed animals relative to their non-alcohol-exposed counterparts.

### Reinforcement Learning

Given the lack of evidence for a general perturbation in subjective reward evaluation, we next used a Pavlovian conditioned approach task to test the effects of adolescent alcohol exposure on reinforcement learning in a separate cohort of animals (*n* = 21). Two months after termination of alcohol exposure, approach behavior was measured during conditioning with paired presentations of a light/lever cue (8 s) followed by the immediate delivery of sucrose reward. Repeated measures ANOVA revealed a significant, non-linear main effect of training session (*F* [1,19] = 39.49, *P*<0.00001) and an interaction between training session and treatment group (*F* [1,19] = 4.32, *P*<0.05) where alcohol-exposed rats exhibited accelerated learning rates compared to controls (Figure 1C) without a shift in general activity (Figure S1 and S2). Following acquisition, all animals underwent extinction training where lever presentation was no longer followed by reinforcement (Figure 1D). Analysis of conditioned responses revealed that both alcohol-exposed and control animals decreased responding across sessions (*F* [1,19] = 339.08, *P*<0.00001). However, there was no effect of treatment group or an interaction between treatment group and session. Thus, while alcohol-exposed animals acquired stimulus-outcome associations faster than controls, they extinguished at an equivalent rate. Interestingly, recent studies using a reinforcement learning framework suggest that an imbalance in the weighting of gains and losses biases choice in probabilistic reinforcement tasks [21], [22]. Therefore, this pattern of altered reinforcement learning may be a feasible mechanism for altered decision making in individuals with a history of adolescent alcohol use.

To verify that this perturbation to learning is applicable to conditions involving the acquired behavioral responses necessary to influence choice, we next assessed instrumental conditioning. Two months following removal of gelatin, during early adulthood, animals were tested on an instrumental learning task (*n* = 18), where lever-press responses were followed by sucrose reward. Alcohol-exposed animals again demonstrated accelerated acquisition but no difference in extinction learning (Figure 1E and 1F). For acquisition, repeated measures ANOVA revealed a significant, non-linear main effect of training session (*F* [1,16] = 27.75, *P*<0.0001) and an interaction between training session and treatment group (*F* [1,16] = 4.93, *P*<0.05). For extinction, analysis of conditioned responses revealed a significant main effect of session (*F* [1,16] = 521.44, *P*<0.00001) but neither an effect of treatment group nor an interaction between treatment group and session. Consistent with these analyses, when we fit the behavioral data using a reinforcement learning model, we observed significantly higher learning rates in alcohol-exposed animals for both Pavlovian and instrumental acquisition (40–46% increase) but not extinction (Figure S3 and S4). We found that separate model fits for the two treatment groups account for the data better than a single model fit for both groups (Pavlovian: *F* [2,248] = 6.69, *P*<0.005, R-squared = 0.46 for alcohol, 0.57 for control; instrumental: *F* [2,366] = 5.46, *P*<0.005, R-squared = 0.57 for alcohol, 0.40 for control). The best fit values from the instrumental task for *α* are 0.312±0.026 for alcohol-exposed rats and 0.222±0.026 for control rats (*t* [15] = 2.42, *P*<0.05). The best fit values from the Pavlovian task for *α* are 0.221±0.028 for alcohol-exposed rats and 0.151±0.012 for control rats (*t* [19] = 2.35, *P*<0.05). Contrary to the results with acquisition, we found that a single model fit the extinction data from both tasks better than separate models (Pavlovian: R-squared = 0.65 for alcohol, 0.59 for control; instrumental: R-squared = 0.65 for alcohol, 0.69 for control). Importantly, reaction times for lever pressing at asymptotic performance were not different between groups in either Pavlovian (*t* [19] = 0.328, *P*<0.05) or instrumental (*t* [15] = 1.091, *P*<0.05) conditioning tasks. Therefore, alcohol-exposed animals exhibited a common learning phenotype during Pavlovian and instrumental conditioning with accelerated positive learning rates, but normal negative learning rates.

### Modeling Action Values and Choice in a Probabilistic Decision-Making Task

To determine whether this disrupted learning in alcohol-exposed animals could contribute to the observed divergence in choice behavior under risk between these animals and controls, we tested animals on a probabilistic decision-making task (*n* = 12) and used a reinforcement learning model to simulate the valuation of instrumental actions. Our goal was to generate a simple model with few parameters to capture the observed behavior, therefore we applied a well-characterized model of reinforcement learning which has been previously shown to capture the slow increase in performance characteristic of acquisition and the slow decrease in performance characteristic of extinction where cues are no longer followed by reward [23], [24]; however, see [25] for an alternative formulation of the extinction learning process. Learning rates from acquisition (*α_pos_*) and extinction (*α_neg_*) were used as estimates of how action values (*Q*) are updated with each positive and negative outcome encountered.

First, we generated a utility function using break points from the progressive ratio task. The utility for each quantity of pellets was calculated as the mean break point for that quantity normalized to the break point for one sucrose pellet from the entire population of rats in the study (Figure 1B; inset). For each rat, outcomes on all trials were used to simulate action values (*Q*) for both the “safe” and “risky” option as they changed from trial to trial. Only the experienced value (in forced trials) and chosen value (in choice trials) was updated after the outcome. For each trial (*t*) the number of pellets received was converted to its utility value, and a reward prediction error value (*δ*) was calculated as the difference between the previously learned *Q*-value for the lever (*i*) and the utility of the outcome on each trial (equation 1). The initial value of both options was set to 1 pellet based on asymptotic performance during instrumental training. *Q* was updated with the rule in equation 2.
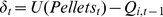
(1)

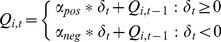
(2)


**Figure 2:**
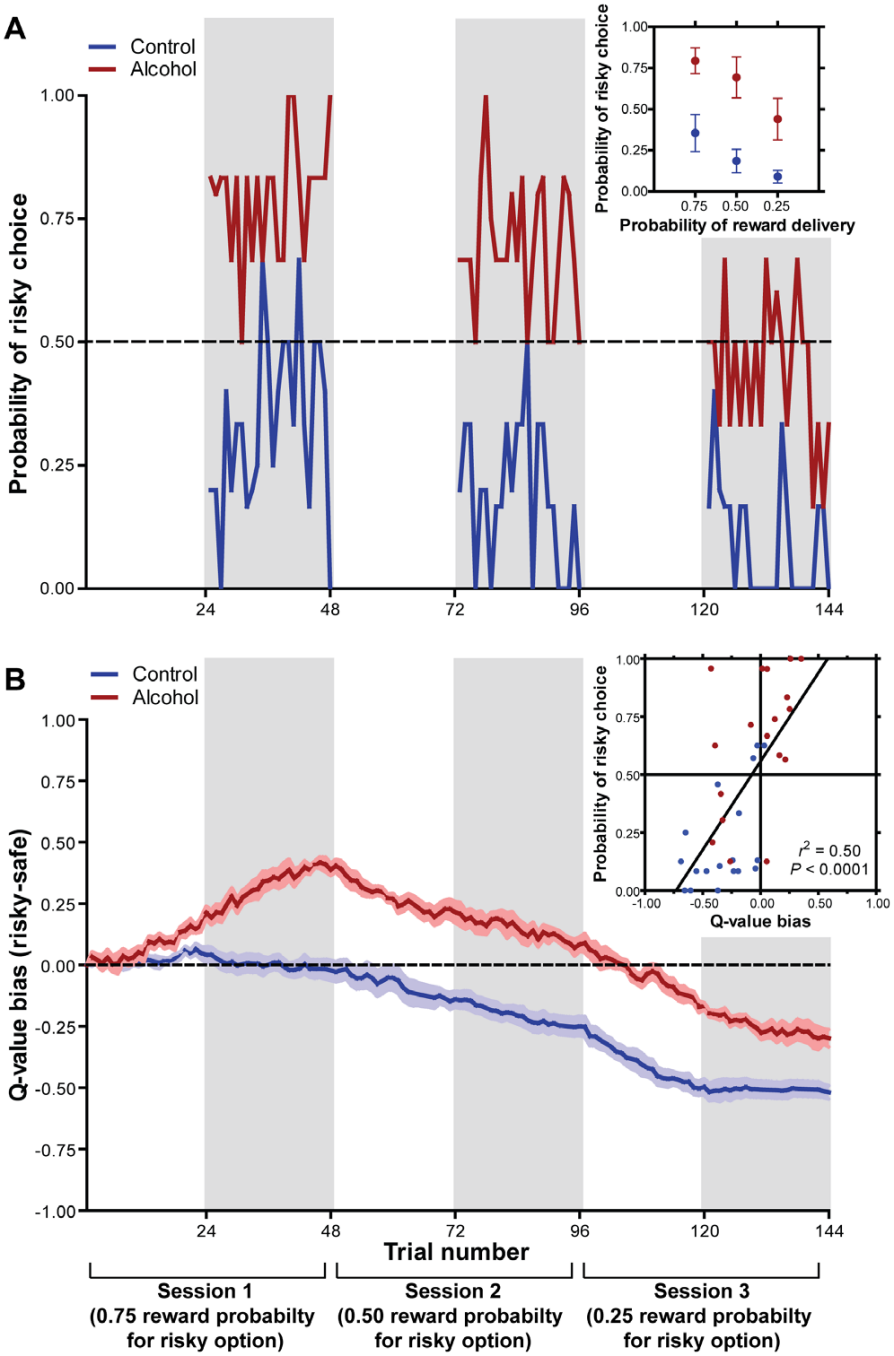
Choice behavior from the probabilistic decision-making task and simulated *Q*-values for alcohol-exposed and control animals. (**A**) Average trial by trial choice of the uncertain option from each probabilistic condition (0.75, 0.50, and 0.25 for delivery of 4 sucrose rewards; gray shading) on the probabilistic decision-making task across sessions in alcohol-treated (red) and control (blue) groups and session average (inset). Probabilistic choice was determined with a concurrent instrumental task involving the presentation of 2 levers, one associated with the certain delivery (probability of 1.00) of 2 sucrose pellets and the other associated with the probabilistic delivery (either 0.75, 0.50, or 0.25) of 4 pellets. During each session, 24 forced-choice trials were followed by 24 free-choice trials (gray shading) with the same probability for the uncertain lever. The forced-choice trials served to expose the rat to each option and its associated expected value. (**B**) Average trial by trial *Q*-values across sessions and correlation to choice behavior (inset). *Q*-values during choice trials are shaded in gray. All data are presented as mean ± SEM.

The learning rates (*α*) were derived from curve fits (Figure S5) of data obtained during acquisition of instrumental responses and determined by the treatment group of the animal and the sign of the reward prediction error (Table S1). We then calculated a *Q*-value bias score for each rat on each trial as the difference between *Q_t_* for the risky and safe levers. Trial-by-trial choice behavior significantly differed between treatment groups across probabilistic outcomes (*F* [1,30] = 29.63, *P*<0.0001), as previously described (Figure 2A; [20]). Individual post-hoc tests between groups revealed a significant effect of alcohol exposure for each probabilistic condition (75%: *t* [10] = 3.19, *P*<0.05; 50%: *t* [10] = 3.55, *P*<0.005; 25%: *t* [10] = 2.66, *P*<0.05). The mean *Q*-value bias scores began to diverge between the alcohol-exposed and control groups during the block of forced trials in the first session (Figure 2B). The bias for both groups drifted toward the safe lever for the remainder of trials in parallel with the decrease in value of the probabilistic option. Notably, the difference between groups remained robust throughout all sessions over all probabilistic values, mirroring the observed choice behavior. Indeed, mean *Q*-value bias scores for choice trial blocks were positively correlated with the probability of risky choices within each block (Figure 2A; inset, r = 0.669).

While this simple linear comparison between the modeled *Q*-values and behavior demonstrates a significant relationship, it is assumed that animals apply a decision rule for choice. Therefore we evaluated the addition of a psychometric decision function to the reinforcement learning rule and utility function used to generate the *Q*-values [26]. The soft maximization (softmax) function (equation 3) fit the data better than epsilon greedy (e-greedy) or matching policies as assessed by computing the log-likelihood ratio compared to random choices ([27], Table S2).
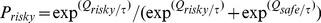
(3)


**Figure 3:**
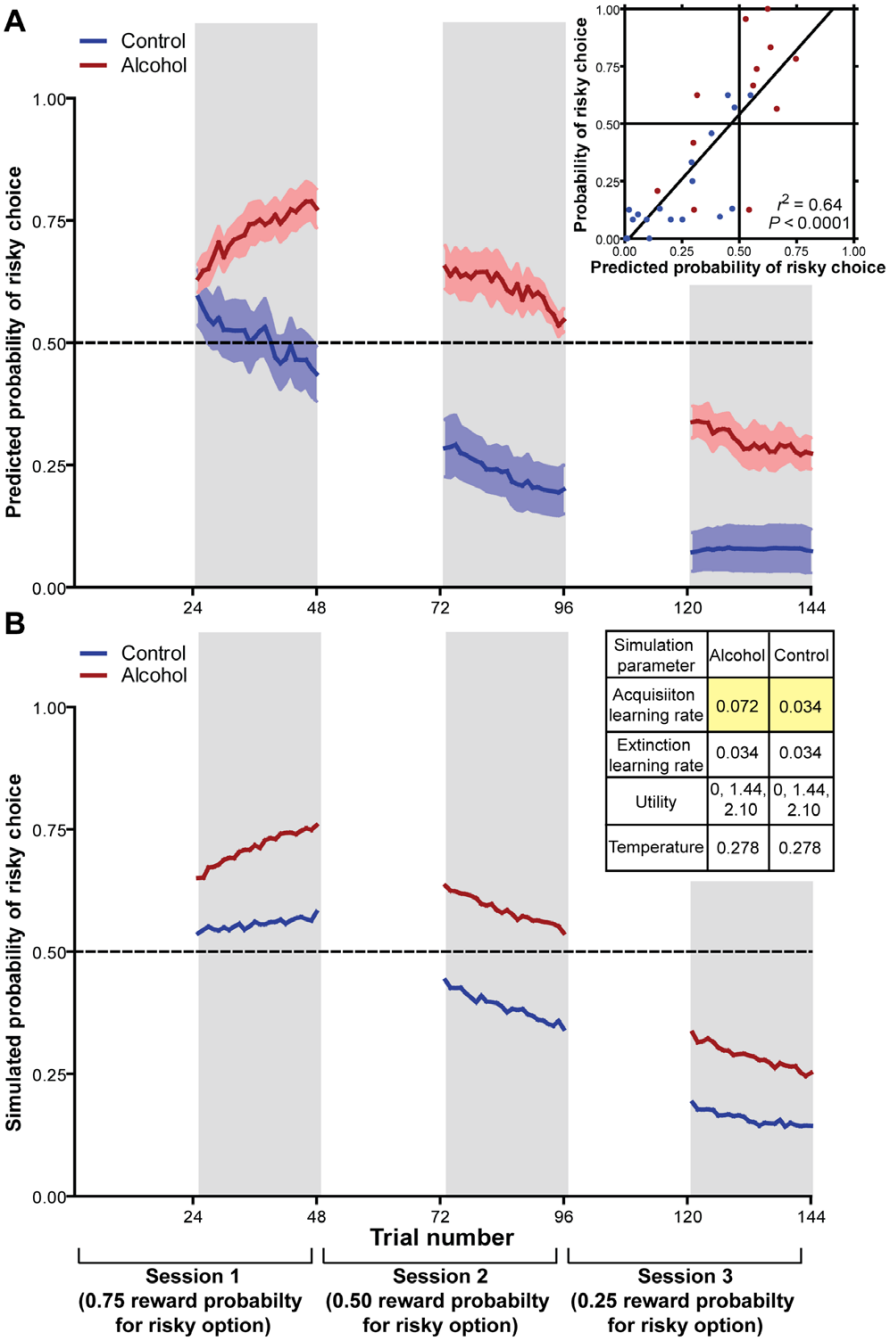
Choice behavior on the probabilistic decision-making task predicted by the softmax function and simulated choice behavior with all parameters except positive learning rate held constant between groups. (**A**) Average trial by trial choice of the uncertain option from each probabilistic condition (0.75, 0.50, and 0.25; gray shading) predicted by the softmax decision function for alcohol-treated (red) and control (blue) groups and correlation with actual choice behavior in these animals (inset). (**B**) Simulated trial by trial choice of the uncertain option from each probabilistic condition (0.75, 0.50, and 0.25; gray shading) using the softmax decision function with all parameters except positive learning rate held constant between groups (inset).

In the softmax model, *τ* is the only free parameter and determines the weight that the difference in *Q*-values is afforded to the rats' choices. As *τ* approaches 0, the rats adopt a greedy maximization policy, in which they always choose the option with the greater *Q*-value. As *τ* approaches ∞, the rats' choices approach randomness with respect to the difference in *Q*-values. For each possible value of *τ* from 0.002 to 10 in increments of 0.002, we calculated the log likelihood of each rat's set of choices as the natural log of the product of the probabilities of each choice made by the rat. Our choice models predicted all 6 control rats (*τ* = 0.213±0.078) and 5 out of 6 alcohol-exposed rats (*τ* = 0.357±0.071) significantly better than chance (Table S2). Importantly, there was no significant effect of alcohol pre-exposure on the best fit value of *τ* (*t* [9] = 1.339, *P*>0.05]. We plotted the predicted probability of risky choice vs. choice trials for each rat's best fitting softmax model, as well as the group means. As expected, the predicted behavior followed the same pattern as the rats' probability of risky choices by session as well as the estimates of *Q*-value bias (Figure 3A). Our model predictions were more strongly correlated with the rats' behavior (r = 0.798) than estimated *Q*-Value bias alone (r = 0.718), confirming that the addition of a decision-making policy did indeed improve modeling of the behavioral data.

### Simulation of Choice Behavior

All of the modeled data presented to this point used the outcomes from the rats' actual decisions to update *Q*-values. However, to be credible, the model should be able to act autonomously, using the outcomes of *its own* decisions to update the *Q*-values. This approach is a much more rigorous test of the model as “errors” carry over to future decisions. Therefore, we ran a simulated experiment to test the effect of learning rate alone on decision-making behavior. We ran 10,000 replicates of two simulations using the model parameters (utility function, decision policy, positive learning rate, and negative learning rate) gleaned from the alcohol-exposed and control animals. The only modeled parameter that differed between the two simulations was the learning rate for better-than-expected outcomes (Figure 3B; inset). In each run of the simulation, a “subject” received alternating blocks of 24 forced and 24 choice trials where one lever was assigned an outcome of 4 pellets with a probability that decreased from 0.75 in the first 48 trials to 0.50 in the next 48 trials, to 0.25 in the last 48 trials, and the other lever was assigned a fixed outcome of 2 pellets, just as in the behavioral experiments in real animals. Our simulated data reproduced the pattern of behavior we observed in rats (Figure 3B). On average, groups gradually decreased their risky choices as the probability of reward decreased, but the group difference remained throughout all 3 sets of choice trials. The simulations demonstrate that an enhanced learning rate for better-than-expected outcomes can, alone, produce differences in choice behavior. Importantly, the divergence in choice behavior conferred solely by the imbalanced learning rate in the simulation is not dependent upon the order that the probabilities are experienced (Figure S6A and S6B). Adolescent alcohol exposure therefore has the potential to profoundly impact an individual's choice behavior under risk through modulation of the reinforcement learning process that determines the values of available options.

## Discussion

It has been recognized for some time that early drug use can be antecedent to substance abuse problems in adulthood. Adolescence is a critical period of maturation where brain development may be disrupted by alcohol use [28]. Cortical and limbic structures, including mesolimbic dopamine circuitry, have been demonstrated to undergo an active developmental period during adolescence that is sensitive to chronic alcohol exposure [29], [30]. A relatively recent consideration is that such experience may also have durable effects on learning and decision-making processes [31], [32] and the specific mechanisms connecting these concepts are largely unresolved. We have shown previously that prior alcohol exposure causally increases risk preference in rats. To identify the fundamental processes underlying the shift in choice behavior produced by adolescent alcohol use, we examined subjective valuation and reinforcement learning. Risk attitude is commonly considered to be a consequence of individual utility functions; however, our behavioral assay of subjective reward valuation under deterministic conditions did not significantly differ in animals with adolescent alcohol intake. The firing rate of midbrain dopamine neurons has been shown to scale with reward magnitude [33] and has been postulated to represent a neural valuation signal [34]. We have previously demonstrated that the encoding of deterministic rewards by this neural valuation signal, phasic dopamine release in the nucleus accumbens, was also unaffected by prior adolescent alcohol use [20], lending further support to the claim that differences in general reward valuation do not account for the altered choice behavior in these animals relative to controls. Interestingly, this dopamine signal only differed in its response to cues predicting probabilistic rewards, where it was elevated, mirroring the behavior preference for these rewards. *Q*-values for predictive stimuli evolve through feedback from reward outcomes during reinforcement learning and are thought to be transmitted by dopamine on cue presentation [35]. Therefore, elevated dopamine release is indicative of increased *Q*-values for probabilistic rewards implicating altered reinforcement learning. Indeed, we observed over-fast learning for better-than-expected, but not worse-than-expected outcomes in rats that with adolescent alcohol exposure. This imbalance in updating the value of an option after positive and negative outcomes is consistent with theoretical work linking altered learning rates from appetitive and aversive outcomes with addiction liability [36] and a recent report by Niv et al. where a reinforcement learning model sensitive to variance in reward was shown to account for individual differences in risk preference [37]. Thus, we used a reinforcement learning model to simulate decision-making behavior following alcohol exposure using four parameters, all of which were obtained from best fit values from the animals' actual behavior. Importantly, the only parameter that differed between groups was the positive learning rate and deviation in this parameter alone was sufficient to recapitulate the behavioral differences between adolescent alcohol-exposed and control rats. We conclude that a specific perturbation in reinforcement learning following adolescent alcohol use can explain altered decision making in subsequent life. These data provide an empirical link between chronic drug use, reinforcement learning and compromised decision making and suggest that altered risk attitude may result from an alteration to a fundamental learning process that impacts many components of behavior.

## Methods

### Animal Subjects

Sixty male Sprague Dawley rats (Charles River; Hollister, CA; obtained at PND 20) were used for experiments. For all experiments, adolescent (PND 30–49) rats [38] were provided with continuous access to a 10% EtOH (*n* = 29) or control (*n* = 31) gelatin for twenty days [7]. Behavioral measures began two months after complete cessation of alcohol access. Prior to all tasks, rats were food deprived to ∼90% of their free-feeding body weight. All rats were housed individually on a 12 hr light-dark cycle with Teklad rodent chow and water available *ad lib* except as noted. Animals were weighed and handled daily. All experimental procedures were in accordance with the Institutional Animal Care and Use Committee at the University of Washington. Some animals (*n* = 8) representing a subset of the probabilistic-choice data set were used in a previous study for separate analyses.

### Alcohol Preparation and Administration

Alcohol was presented to rats in a gel comprised of distilled water, Knox© gelatin, Polycose (10%), and EtOH (10%). This gelatin was made available 24 hrs/day for 20 days in addition to standard chow and water. Preparation followed the methodology of Nasrallah et al., 2009. This procedure was designed to minimize evaporation of ethanol and has been validated to yield accurate ethanol content [39] and to promote alterations in brain chemistry [40]. Experiments began with 3 days of pre-exposure to a control gelatin, and all animals were matched by weight and baseline intake and split into 2 conditions–one group receiving 24 hr access to an alcohol gelatin and the other a control gelatin for 20 days. Mean daily adolescent alcohol intakes prior to the progressive ratio and probabilistic decision-making tasks, instrumental learning task, and Pavlovian conditioned approach task were 7.55 g/kg/day±0.34, 6.17 g/kg/day±0.28, and 6.94 g/kg/day±0.34, respectively.

### Instrumental Learning and Extinction

Rats (*n* = 17) first underwent magazine training, in which they were given 10 minutes in a standard operant chamber (Med Associates, VT) to consume 45 mg sucrose pellets (Bio-Serve, NJ) in the magazine tray. Next, rats were tested on 8 daily sessions of a discrete-trial instrumental learning task. Each session consisted of 30 trials, presented on a variable 45 second inter-trail interval, with a single session per day. During a given trial, one lever was extended for 8 seconds. A lever response was reinforced with delivery of a single 45 mg sucrose pellet delivered in the magazine tray and followed by retraction of the lever. Following acquisition, both groups underwent 15 extinction sessions where the lever press response was followed by retraction of the lever but no reinforcement. A separate group of rats (*n* = 10) were run on a control task, where lever presses were never followed by reward delivery, to assess any differences in activity specific to the conditioning environment between the two groups. Number of lever presses and latency to lever press were recorded throughout for subsequent analysis with mixed measures ANOVA with session as a repeated measure and treatment group as a between groups measure.

To model the acquisition of lever pressing behavior in response to reinforcement, we binned trials into groups of ten (decades) and took the number of responses per decade divided by 10 as an estimate of the response probability per trial for that decade. We then assumed that response probability in decade n (P_n_) is proportional to the associative strength of the CS and thus would behave according to a standard reinforcement learning model [23] of conditioning (equations 4 and 5). Therefore, we fit the data with an exponential equation (6), the analytic solution to equations 4 and 5 when P0 = 0. P_max_ is the response probability at asymptote, and α is the learning rate per decade. Both P_max_ and α are free parameters that vary between 0 and 1,
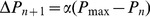
(4)

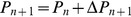
(5)

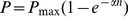
(6)


### Pavlovian Conditioning and Extinction

Rats (*n* = 21) first underwent magazine training in which they were given 10 minutes in a standard operant chamber to consume 45 mg sucrose pellets in the magazine tray. Rats then received 5 daily sessions of a discrete-trial Pavlovian conditioned approach task. Each session consisted of 25 trials, presented on a variable 60 second inter-trial interval, with a single session per day. A trial consisted of a lever/light cue presented for 8 seconds followed immediately by the non-contingent delivery of a single 45 mg sucrose pellet delivered in the magazine tray and retraction of the lever. Lever presses were recorded but were without consequence for reward delivery. Following acquisition both groups underwent 5 extinction sessions where lever presentation was not followed by reward delivery. Number of lever presses and latency to lever press were recorded throughout for subsequent analysis with mixed measures ANOVA with session as a repeated measure and treatment group as a between groups measure. Acquisition was modeled as described above for instrumental conditioning.

### Progressive Ratio

The progressive-ratio task was conducted in a standard operant chamber. Prior to the progressive-ratio task, all rats (*n* = 12) were trained to lever press for a single 45 mg sucrose pellet until they met a criterion of >80% responses within a session. The progressive-ratio task consisted of three sessions where the reward magnitude was 1, 2, or 4 pellets. The order of session (ascending- 1, 2, 4 pellets or descending 4, 2, 1 pellets) was counterbalanced within treatment groups. During a session, the response requirement for reward delivery increased across trials by a multiplicative factor of the square root of 2. Failure to reach the response requirement within 15 minutes led to the termination of the session. This point is defined as the subject's “break point” and is quantified as the number of responses in the last completed trial. To assess whether any differences between treatments groups or across reward magnitude was attributable to differences in satiation, a 15 minute free-operant (FR1) task was run immediately following each session of the progressive- ratio task. Rate of lever pressing did not differ between groups or across reward values suggesting that satiety did not contribute to the amount of effort exerted to obtain food reward at different values (main effect of value: *F* [2,20] = 2.02, *P*>0.05; main effect of treatment group: *F* [1,20] = 1.77, *P*>0.05; interaction effect: *F* [2,20] = 0.22 *P*>0.05). Break point data were analyzed with mixed measures ANOVA with reward magnitude as a repeated measure and treatment group as a between groups measure. For use in modeling utility functions, break points were normalized for each rat by the break point for 1 food pellet and the utility of 0 pellets was assumed to be 0.

### Probabilistic Decision-Making Task

Rats (*n* = 12) first underwent magazine training in which they were given 10 minutes in a standard operant chamber to consume 45 mg sucrose pellets in the magazine tray. Rats were then trained on a fixed-ratio 1 discrete-operant schedule (FR1) for 45 mg sucrose pellets on both levers to a criterion of 80% response rate in a session (30 trials per session). This was followed by a concurrent instrumental task involving the presentation of 2 levers, one associated with the certain delivery (probability of 1.00) of 2 sucrose pellets and the other associated with the probabilistic delivery (0.75, 0.50, or 0.25 presented in descending order in three separate sessions) of 4 pellets. Each daily 45 min session consisted of 24 forced trials followed by 24 free-choice trials. At the start of each session, the chamber was in the inter-trial interval state–completely dark with no light cues. All trials began with illumination of the house light and a light in the food tray cueing the rat to make a nose-poke into the food tray within 10 sec. This ensured that the subject was centered in the chamber at the start of each trial, eliminating position bias. Failure to nose-poke resulted in trial termination, and the chamber returned to the inter-trial interval state. During training, rats were exposed to forced trials wherein a successful nose-poke led to the extension of a single lever, presented pseudo-randomly. A response was required within 10 seconds or the trial was terminated and the chamber returned to the inter-trial interval. A successful response resulted in the illumination of the tray light and delivery of reward, based on the associated probability, followed by an inter-trial interval of 45 sec. Forced trial sessions consisted of 24 trials. These trials served to expose the rat to each option and its associated expected value.

During each session, forced choice trials were followed by free-choice trials with the same probability for the uncertain lever. Free-choice trials followed the guidelines described above, but each successful nose-poke resulted in the extension of both levers, and the rat was free to choose between the two levers within 10 sec. Thus, this session offered the rat a choice between the 2 levers to assess the rat's preference between options. Lever choice was recorded and analyzed using repeated measures ANOVA, with probability as repeated measure and alcohol treatment as the between group measure.

### Modeling

We evaluated 3 different decision functions: soft maximization (softmax), epsilon greedy (e-greedy), and matching. The softmax function is described by (equation 3), in which *τ* is a free parameter that determines the randomness of the rats' choices. As *τ* approaches 0, the rats adopt a greedy maximization policy, in which they always choose the option with the greater *Q*-value. As *τ* approaches ∞, the rats' choices are completely random regardless of the difference in *Q*-values.

The e-greedy policy (simplified here for a two-choice task) predicts that the rat will chose to exploit the greater option with the greater *Q*-Value with a probability of 1-ε, and will explore the lesser option with a probability equal to ε.
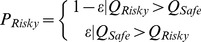
(7)The matching policy predicts the rat will chose each option with a probability equal to the proportion of total reward available.
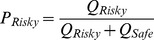
(8)For all models:
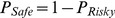
(9)We calculated a likelihood score for all 3 behavioral policies for each rat. For the softmax policy we allowed *τ* to vary from 0.002 to 10 in increments of 0.002. For the e-greedy policy, we allowed *ε* to vary from 0 to 0.5 in increments of 0.0001. The matching policy does not require any parameter fitting. For each possible *τ* and ε we calculated the log likelihood of each rat's set of choices as the natural log of the product of the probabilities of each choice made by the rat. Probabilities for missed choice trials were not included in the models. We then computed the log likelihood ratio for each softmax and e-greedy fit with respect to completely random choices (*P_c_* = 0.5 for all *c*) and matching, and used the chi-squared distribution (df = 1) to convert likelihood ratios to P values. A similar comparison could not be used between softmax and greedy epsilon models because they contain the same number of free parameters and therefore cannot be treated as nested models.

### Behavioral Simulation

We ran two sets of 10000 simulations, and the sets only differed by the learning rate for positive prediction errors as between alcohol exposed and control groups. In each run of the simulation, a subject received alternating blocks of 24 forced and 24 choice trials. One lever was assigned an outcome of 4 pellets with a probability that decreased from 0.75 in the first 48 trials to 0.50 in the next 48 trials, to 0.25 in the last 48 trials. The other lever was assigned a fixed outcome of 2 pellets. We generated action values for both levers using the same utility function and reinforcement-learning model as in our action-value simulations. Behavior on choice trials and outcomes of probabilistic levers were determined by the Matlab rand function using the Mersenne Twister algorithm. The probability of risky choice was determined using the softmax function (equation 4) with *τ* = 0.278, which was the average value for all significantly performing softmax models from our behavior fits.

## Supporting Information

Figure S1 **Non-reinforced lever pressing in alcohol-exposed and control animals.** To control for differences in generalized activity, which could potentially account for differences in learning, we measured lever pressing behavior in a non-reinforced variant of the instrumental task (*n* = 10). Non-reinforced lever-pressing behavior did not significantly differ between alcohol-treated (red) and control (blue) groups. Data are presented as mean ± SEM.
(TIF)

Figure S2 **Locomotor activity in alcohol-exposed and control animals.** Horizontal locomotor activity was measured in an open field chamber equipped with photobeam rings (Truscan chamber 40.6×40.6×40.6 cm, Coulbourn Instruments, Allentown, PA). X–Y coordinates, obtained at a sample rate of 1/s, were used to determine the rat's position in the chamber. Distance and time traveled were calculated by summing the sequential changes in position obtained from the coordinates throughout a 20 minute session. Distance traveled before the Pavlovian conditioning task (**A**) and the unpaired instrumental task (**B**) for alcohol-treated (red) and control (blue) rats did not significantly differ between treatment groups (prior to Pavlovian task: *t*[19] = 0.32, *P*>0.05; prior to instrumental control: *t*[8] = 1.09, *P*>0.05). Data are presented as mean ± SEM.
(TIF)

Figure S3 **Acquisition data from the Pavlovian and instrumental conditioning tasks.** The data from Pavlovian (**A**) and instrumental (**B**) conditioning are binned into decades and fit to a standard Reinforcement Learning model for comparison between alcohol-treated (red) and control (blue) groups. Data are presented as mean ± SEM.
(TIF)

Figure S4 **Extinction data from the Pavlovian and instrumental conditioning tasks.** The data from Pavlovian conditioning (**A**) and instrumental conditioning (**B**) are binned into decades and fit to a standard Reinforcement Learning model for comparison between alcohol-treated (red) and control (blue) groups. Data are presented as mean ± SEM.
(TIF)

Figure S5 **Acquisition data during instrumental training for the adolescent rats used in the probabilistic decision-making task.** The data are binned into decades and fit to a standard reinforcement learning model for comparison between alcohol-exposed (red) and control (blue) rats. We found that separate model fits for the two treatment groups account for the data better than a single model fit for both groups (*F* [2,73] = 6.20, *P*<0.005, R-squared = 0.30 for alcohol, 0.22 for control). Data are presented as mean ± SEM.
(TIF)

Figure S6 **Simulated choice behavior on the probabilistic decision-making task with all parameters except positive learning rate held constant between groups.** Simulated trial by trial choice of the uncertain option from each probabilistic condition using the softmax decision function with all parameters except positive learning rate held constant between groups. The simulation was run with the order of probabilistic conditions was reversed (0.25, 0.50, 0.75) in (**A**) and with the 0.50 condition across all three sessions (**B**). These simulations demonstrate that the divergence in choice behavior between groups as a result of an imbalance in learning is robust with respect to the order in which the conditions are experienced.
(TIF)

Table S1 **Learning rates by sign of δ_t_ and treatment group.**
(DOC)

Table S2 **Best fit values of** ***τ*** **and** ***ε*** **for each rat and their respective log likelihoods and P-values when compared to chance and matching.** A higher log likelihood score indicates that a model performed better at predicting the rat's behavior. Best fit values for models that are significantly better than chance are shown in bold.
(DOC)
