# Supplementary figures and images for: Altered Risk-Based Decision Making following Adolescent Alcohol Use Results from an Imbalance in Reinforcement Learning in Rats
Source: PLoS One. 2012 May 16;7(5):e37357. doi: 10.1371/journal.pone.0037357 (PMC3353889; doi:10.1371/journal.pone.0037357)

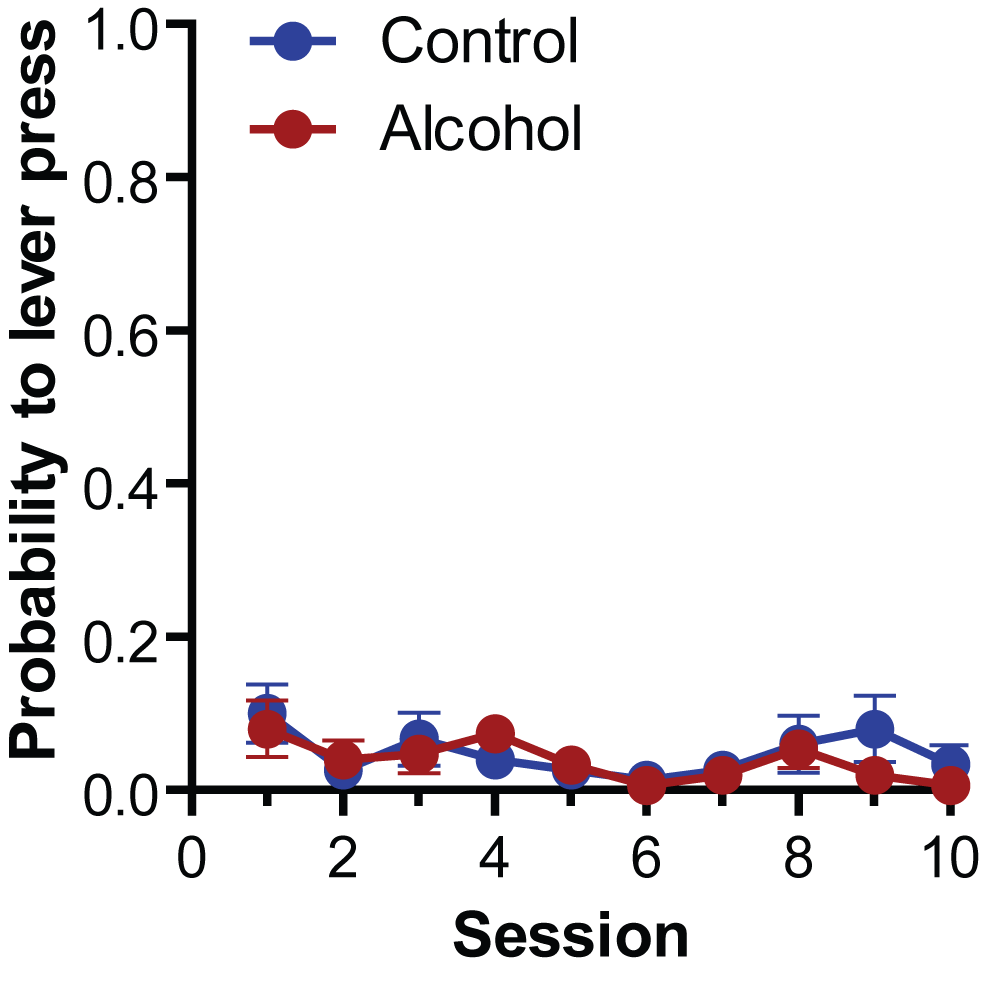

Supplement: Figure S1 — Non-reinforced lever pressing in alcohol-exposed and control animals. To control for differences in generalized activity, which could potentially account for differences in learning, we measured lever pressing behavior in a non-reinforced variant of the instrumental task (n = 10). Non-reinforced lever-pressing behavior did not significantly differ between alcohol-treated (red) and control (blue) groups. Data are presented as mean ± SEM. (TIF) [file pone.0037357.s001.tif]

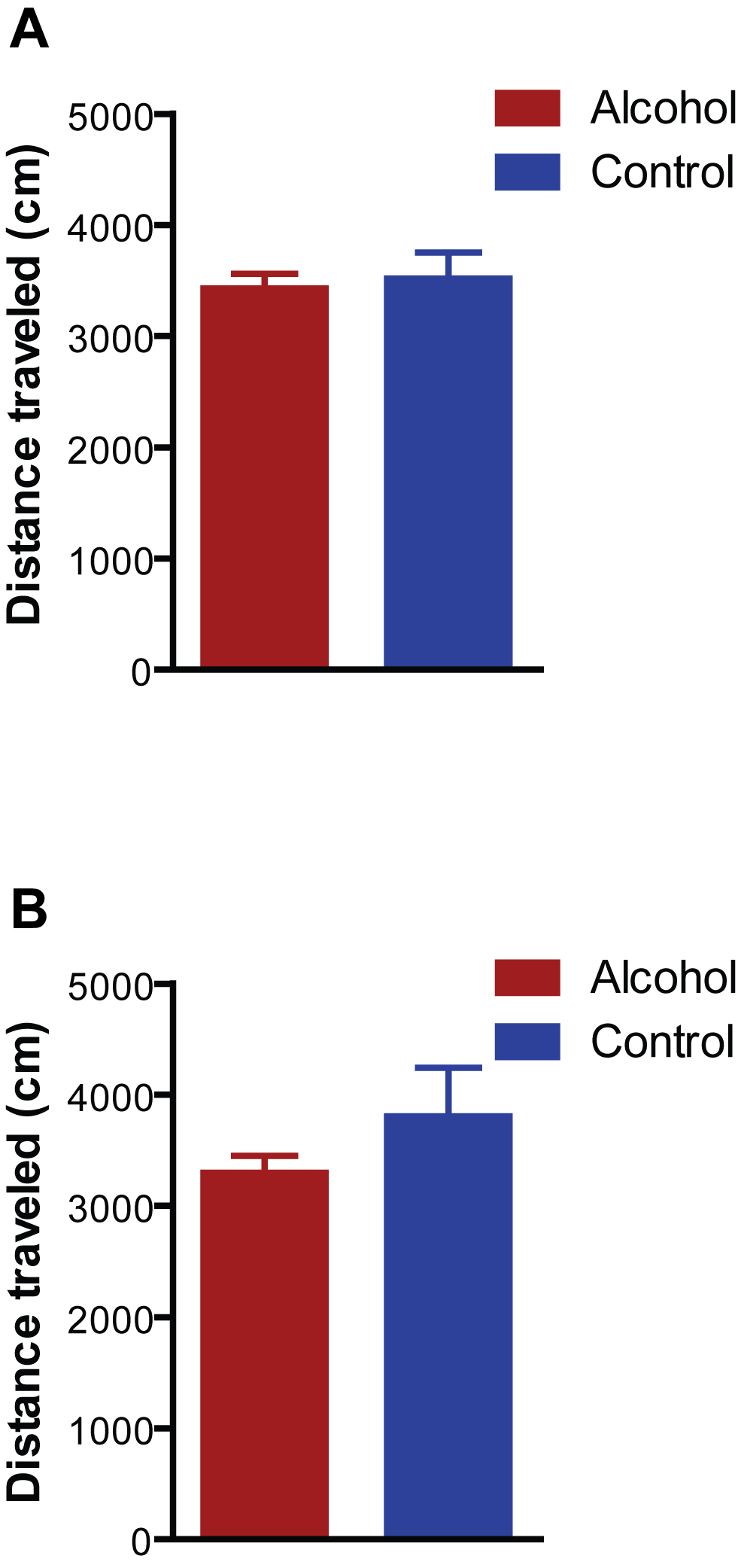

Supplement: Figure S2 — Locomotor activity in alcohol-exposed and control animals. Horizontal locomotor activity was measured in an open field chamber equipped with photobeam rings (Truscan chamber 40.6×40.6×40.6 cm, Coulbourn Instruments, Allentown, PA). X–Y coordinates, obtained at a sample rate of 1/s, were used to determine the rat's position in the chamber. Distance and time traveled were calculated by summing the sequential changes in position obtained from the coordinates throughout a 20 minute session. Distance traveled before the Pavlovian conditioning task (A) and the unpaired instrumental task (B) for alcohol-treated (red) and control (blue) rats did not significantly differ between treatment groups (prior to Pavlovian task: t[19] = 0.32, P>0.05; prior to instrumental control: t[8] = 1.09, P>0.05). Data are presented as mean ± SEM. (TIF) [file pone.0037357.s002.tif]

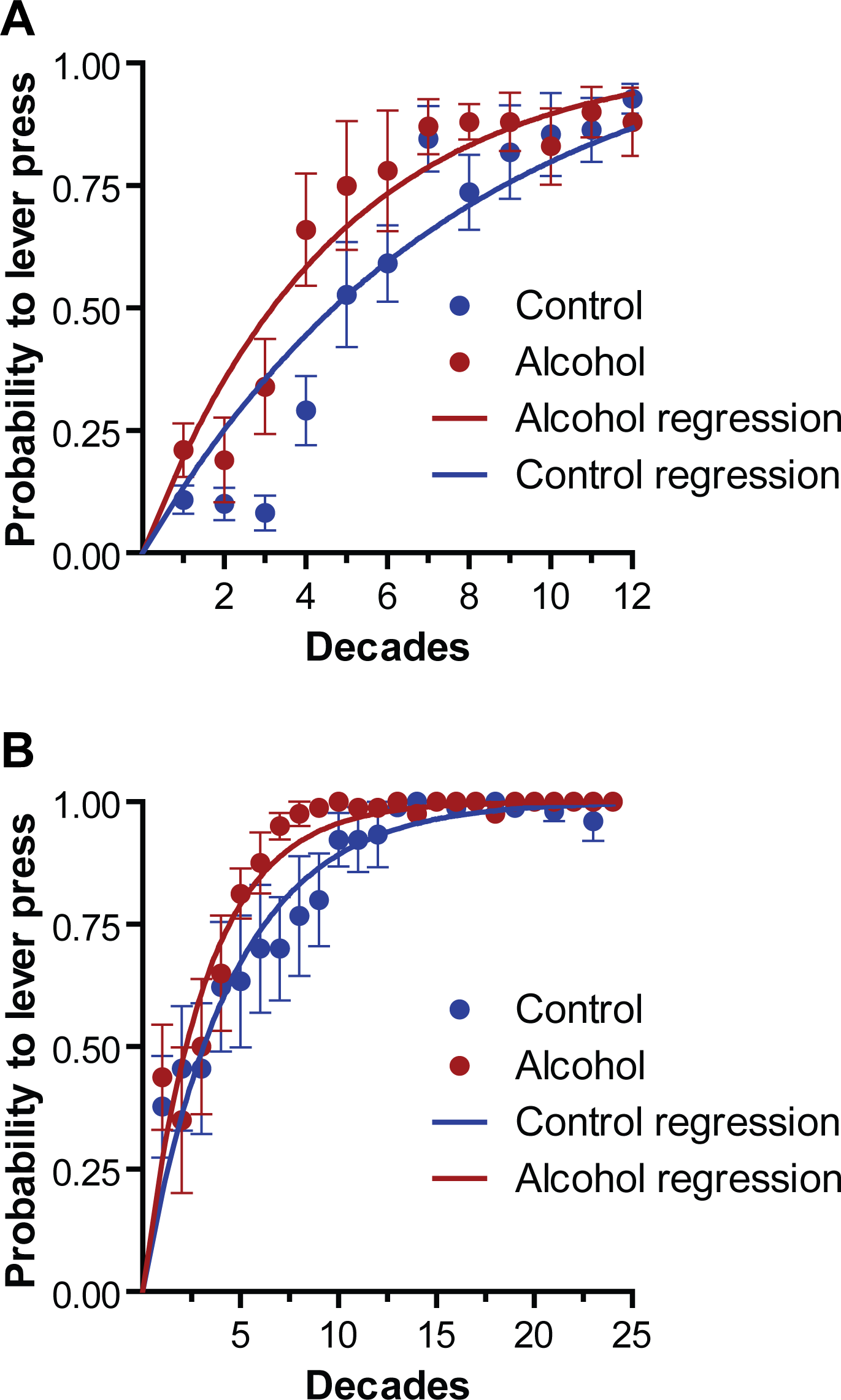

Supplement: Figure S3 — Acquisition data from the Pavlovian and instrumental conditioning tasks. The data from Pavlovian (A) and instrumental (B) conditioning are binned into decades and fit to a standard Reinforcement Learning model for comparison between alcohol-treated (red) and control (blue) groups. Data are presented as mean ± SEM. (TIF) [file pone.0037357.s003.tif]

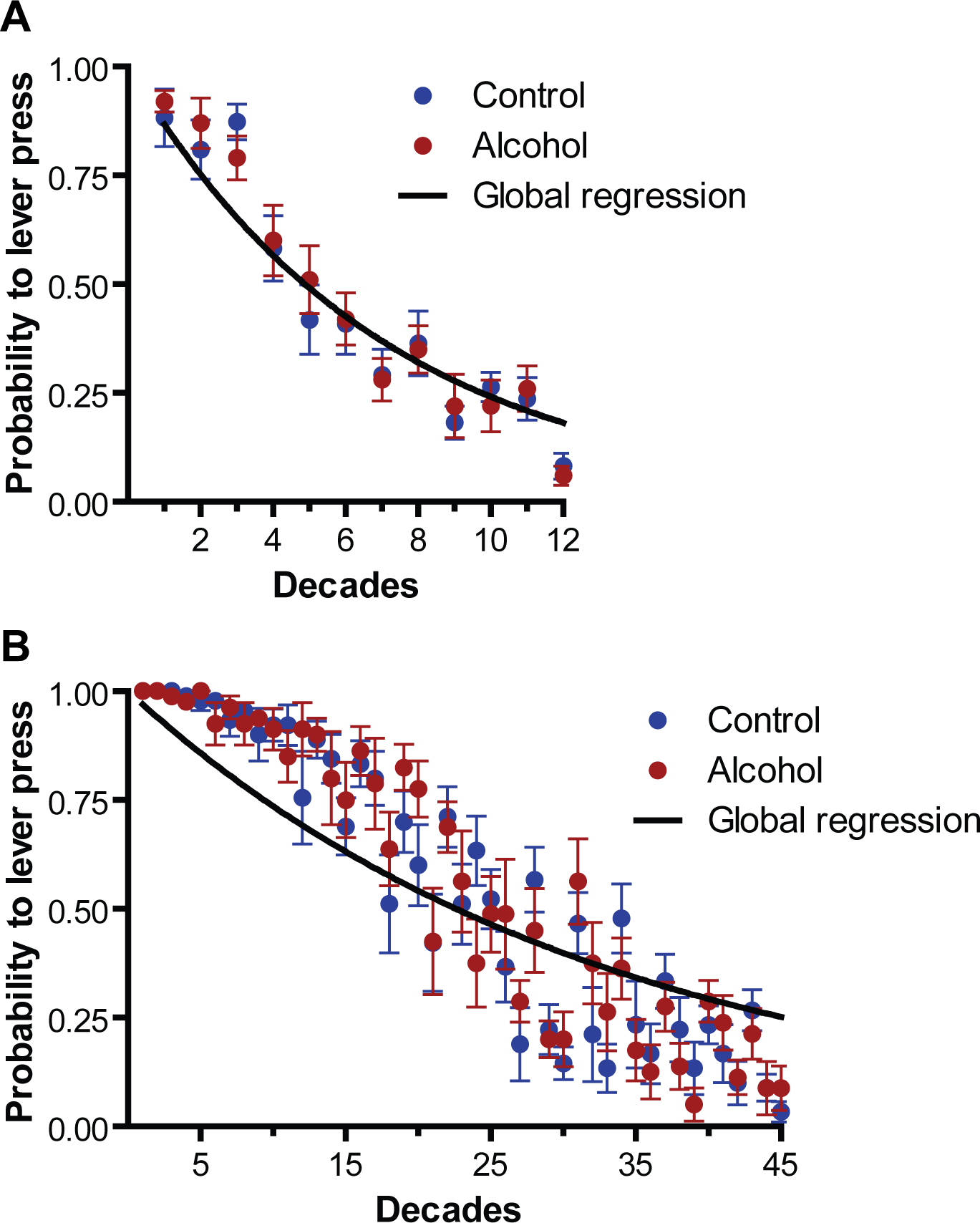

Supplement: Figure S4 — Extinction data from the Pavlovian and instrumental conditioning tasks. The data from Pavlovian conditioning (A) and instrumental conditioning (B) are binned into decades and fit to a standard Reinforcement Learning model for comparison between alcohol-treated (red) and control (blue) groups. Data are presented as mean ± SEM. (TIF) [file pone.0037357.s004.tif]

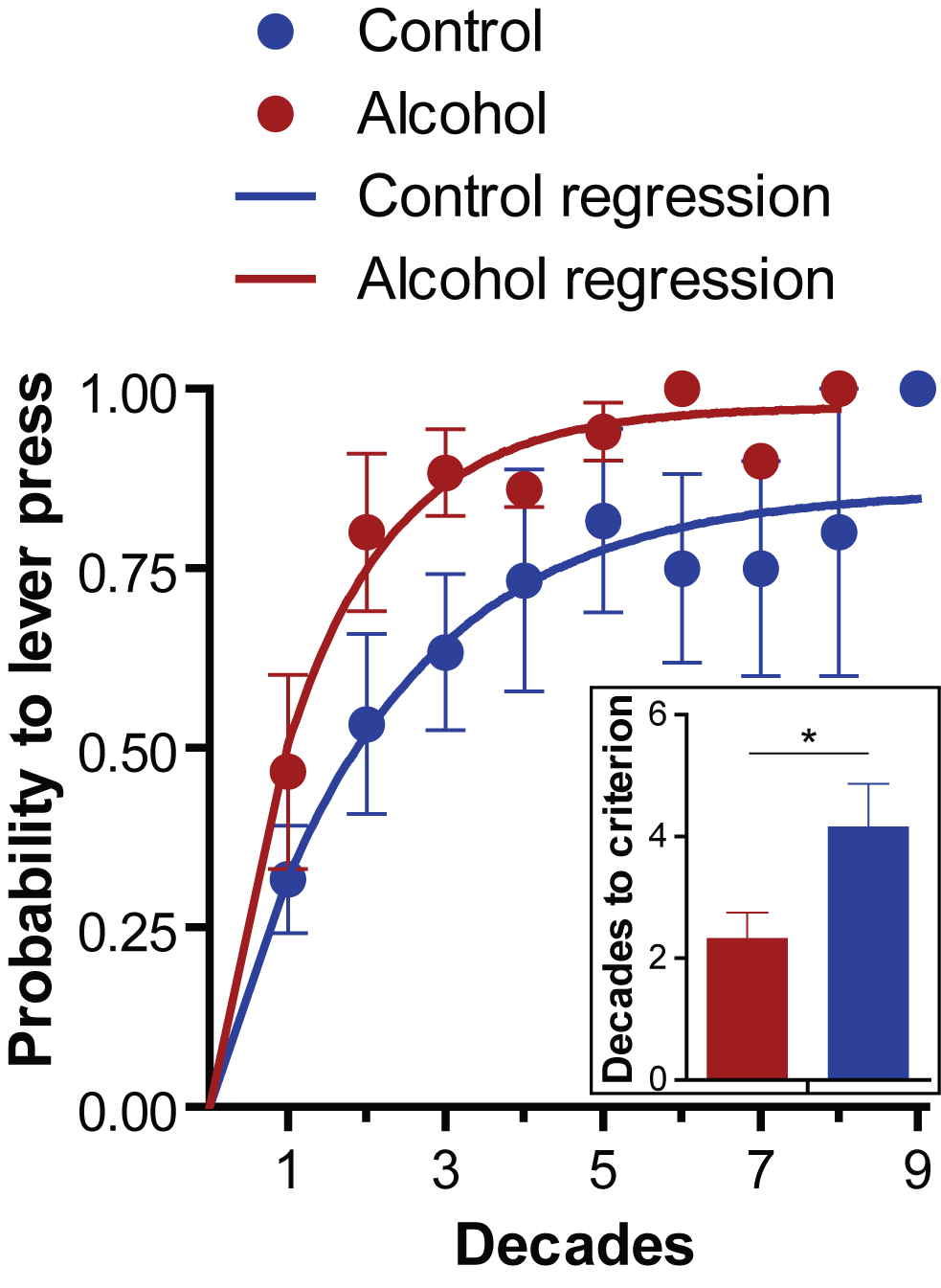

Supplement: Figure S5 — Acquisition data during instrumental training for the adolescent rats used in the probabilistic decision-making task. The data are binned into decades and fit to a standard reinforcement learning model for comparison between alcohol-exposed (red) and control (blue) rats. We found that separate model fits for the two treatment groups account for the data better than a single model fit for both groups (F [2,73] = 6.20, P<0.005, R-squared = 0.30 for alcohol, 0.22 for control). Data are presented as mean ± SEM. (TIF) [file pone.0037357.s005.tif]

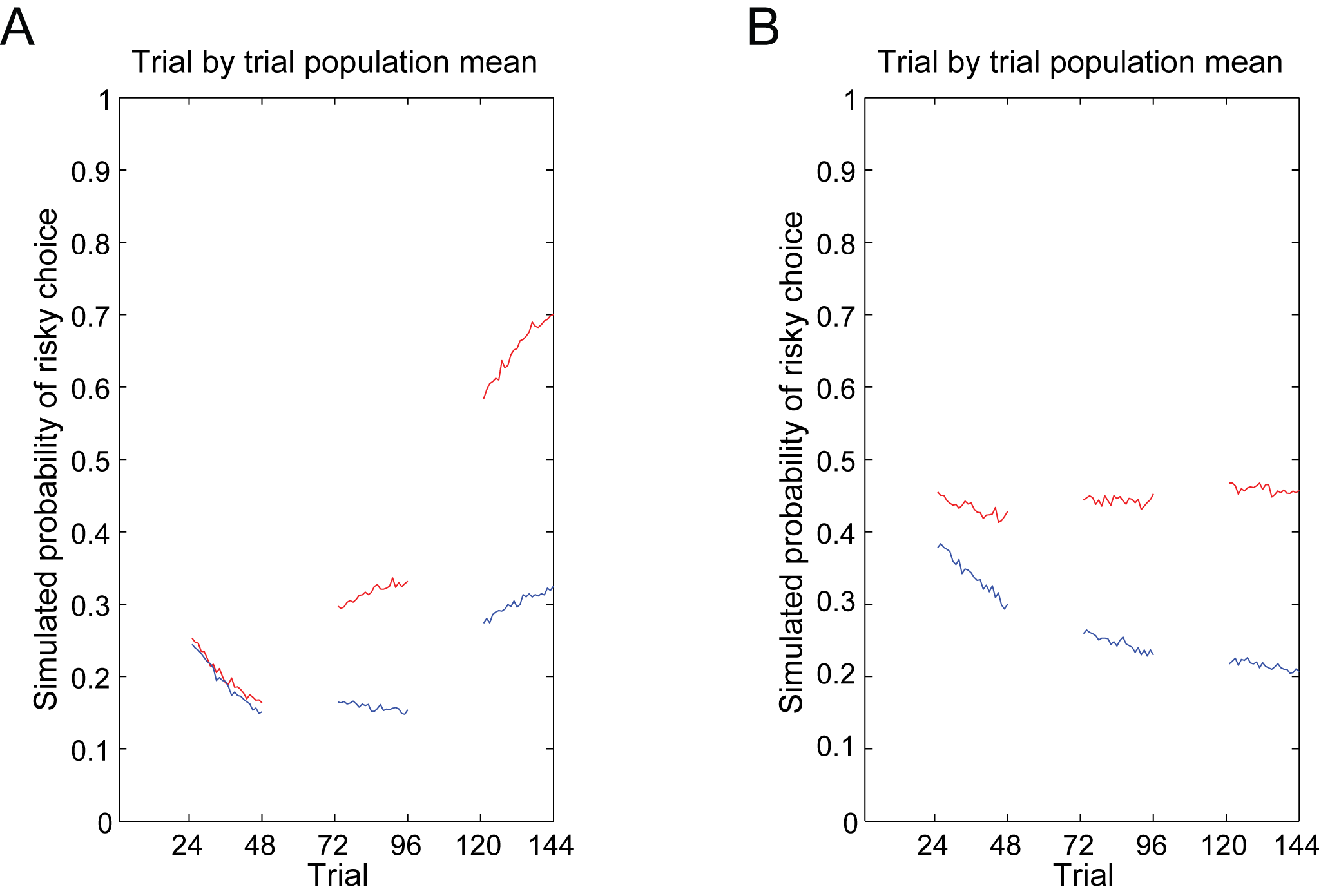

Supplement: Figure S6 — Simulated choice behavior on the probabilistic decision-making task with all parameters except positive learning rate held constant between groups. Simulated trial by trial choice of the uncertain option from each probabilistic condition using the softmax decision function with all parameters except positive learning rate held constant between groups. The simulation was run with the order of probabilistic conditions was reversed (0.25, 0.50, 0.75) in (A) and with the 0.50 condition across all three sessions (B). These simulations demonstrate that the divergence in choice behavior between groups as a result of an imbalance in learning is robust with respect to the order in which the conditions are experienced. (TIF) [file pone.0037357.s006.tif]
