# Supplementary material for: Altered Risk-Based Decision Making following Adolescent Alcohol Use Results from an Imbalance in Reinforcement Learning in Rats
Source: PLoS One. 2012 May 16;7(5):e37357. doi: 10.1371/journal.pone.0037357 (PMC3353889; doi:10.1371/journal.pone.0037357)
Supplement: Table S1 — Learning rates by sign of δt and treatment group. (DOC) [file pone.0037357.s007.doc]

| Group | Positive δ_t_ | Negative δ_t_ |
| --- | --- | --- |
| Control | 0.034 | 0.034 |
| Alcohol Exposed | 0.072 | 0.034 |
