# Supplementary material for: Altered Risk-Based Decision Making following Adolescent Alcohol Use Results from an Imbalance in Reinforcement Learning in Rats
Source: PLoS One. 2012 May 16;7(5):e37357. doi: 10.1371/journal.pone.0037357 (PMC3353889; doi:10.1371/journal.pone.0037357)
Supplement: Table S2 — Best fit values of τ and ε for each rat and their respective log likelihoods and P-values when compared to chance and matching. A higher log likelihood score indicates that a model performed better at predicting the rat's behavior. Best fit values for models that are significantly better than chance are shown in bold. (DOC) [file pone.0037357.s008.doc]

|  | Softmax | | | | e-Greedy | | | | Matching |
| --- | --- | --- | --- | --- | --- | --- | --- | --- | --- |
| Rat | *τ* | Log Likelihood | P vs. Chance | P vs. Matching | *ε* | Log Likelihood | P vs. Chance | P vs.  Matching | Log  Likelihood |
| Control | | | | | | | | | |
| C 1 | **0.146** | -25.5 | <0.0001 | <0.0001 | **0.0694** | -18.2 | <0.0001 | <0.0001 | -42.2 |
| C 2 | **0.090** | -26.1 | <0.0001 | <0.0001 | **0.2239** | -35.6 | <0.0001 | 0.0007 | -41.4 |
| C 3 | **0.138** | -27.6 | <0.0001 | <0.0001 | **0.1563** | -27.7 | <0.0001 | <0.0001 | -39.6 |
| C 4 | **0.184** | -40.3 | <0.0001 | 0.0005 | 0.3944 | -47.6 | 0.0740 | 1 | -46.5 |
| C 5 | **0.124** | -22.2 | <0.0001 | <0.0001 | **0.2344** | -34.8 | <0.0001 | 0.0184 | -37.6 |
| C 6 | **0.598** | -44.7 | 0.0125 | 0.2471 | **0.3768** | -45.7 | 0.0397 | 1 | -45.4 |
| Alcohol Exposed | | | | | | | | | |
| A 1 | **0.382** | -44.9 | 0.0360 | 0.1314 | **0.3676** | -44.7 | 0.0281 | 0.1002 | -46.1 |
| A 2 | **0.224** | -37.9 | <0.0001 | 0.0001 | **0.2254** | -37.9 | <0.0001 | 0.0001 | -45.5 |
| A 3 | **0.616** | -47.5 | 0.0271 | 0.1052 | **0.3611** | -47.1 | 0.0177 | 0.0663 | -48.8 |
| A 4 | **0.246** | -39.6 | <0.0001 | 0.0003 | **0.2254** | -37.9 | <0.0001 | 0.0001 | -46.1 |
| A 5 | **0.316** | -43.4 | 0.0006 | 0.0087 | **0.2817** | -42.2 | 0.0002 | 0.0024 | -46.8 |
| A 6 | 2.966 | -49.2 | 0.7565 | 1 | 0.4789 | -49.2 | 0.7218 | 1 | -49.1 |
